# Inhibition Effect of Coal Spontaneous Combustion by Composite Inhibitory Foam Based on CaCl_2_–Melatonin Inhibitor

**DOI:** 10.1021/acsomega.3c10258

**Published:** 2024-03-06

**Authors:** Hong Zhang, Zongquan Zhao, Zihan Wang, Shibin Nie, Chao Han

**Affiliations:** † College of Safety Science and Engineering, Anhui University of Science and Technology, Huainan, Anhui 232001, P R China; ‡ College of Public Security and Emergency Management, Anhui University of Science and Technology, Hefei, Anhui 231131, P R China; § Institute of Energy, Hefei Comprehensive National Science Center, Hefei, Anhui 230000, P R China

## Abstract

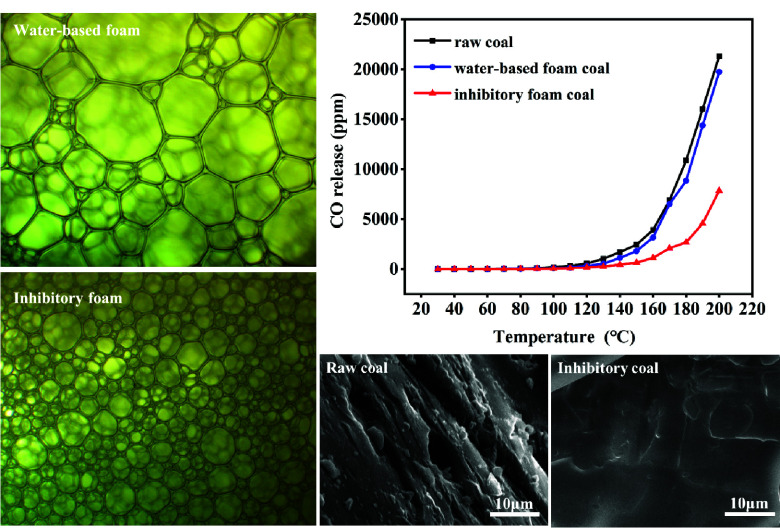

Inhibitory foam technology plays an important role in inhibiting coal spontaneous combustion. To enhance the stability and inhibitory performance of inhibitory foam for coal spontaneous combustion, a novel physicochemical composite inhibitor was developed in this work. CaCl_2_ was chosen as an inorganic salt physical inhibitor to compound with the chemical inhibitor melatonin (MLT) due to its corresponding good foam stability. When the mass ratio of CaCl_2_ to MLT was 4:1, the lowest CO release concentration of 7337.06 ppm at 200 °C was observed in the composite inhibitor-treated coal. Furthermore, the addition of 20 wt % of the composite inhibitor resulted in a foam half-life of 3067 min, which was 5.89 times longer than that of the water-based foam. In comparison with the water-based foam, the inhibitory foam based on 20 wt % CaCl_2_–MLT composite inhibitor exhibited more excellent foam stability, wetting ability, and inhibition performance. The release of CO at 200 °C was 7854.6 ppm, showing a reduction of 63.2% compared to the raw coal. Moreover, the composite inhibitory foam could significantly delay the onset of the characteristic temperature and reduce the weight change during the decomposition stage by 12.8%.

## 1 Introduction

As an important energy source in the world, coal plays an important role in the chemical industry, steel, and power generation.^1−4^ Coal spontaneous combustion, which occurs during coal mining, storage, and transportation, is considered one of the most dangerous natural disasters encountered in the coal industry.^5−7^ To inhibit spontaneous combustion in coal mines, various methods have been proposed by scholars, including grouting, injection of inert gases, foams, inhibitors, and gels.^8−11^ However, these techniques still suffer from many drawbacks, such as the gully phenomenon of grouting,^12^ the strict underground environmental conditions for inert gases,^13^ and the limited diffusion range of gels and inhibitors.^14−16^ Therefore, development of efficient coal mine fire prevention and extinguishing technology is crucial for prevention and control of coal spontaneous combustion.

According to the hypothesis of coal–oxygen complex reactions,^17^ the accumulation of heat caused by the contact between active groups in coal and oxygen is the primary cause of spontaneous combustion in coal. Reducing the contact between coal and oxygen, as well as the generation of free radicals, is the main focal point for addressing the issue of coal spontaneous combustion. The use of inhibitory foam enables the delivery of inhibitors to the closed goaf area and the formation of a fire barrier, which limits the spread of the fire effectively.^18^ It is essential to develop a novel inhibitory foam that combines excellent stability and the inhibition effect of coal spontaneous combustion. The inhibitory performance of the inhibitory foam is greatly influenced by the composition of the inhibitor. The use of physical inhibitors (mainly inorganic salt solutions such as NaCl, CaCl_2_, Na_2_SO_4_, and NaHCO_3_) can effectively isolate oxygen and reduce the surface temperature of coal,^19−22^ which has been proven to be one of the most common ways to inhibit coal spontaneous combustion. However, adding inorganic salt inhibitors generally causes a salting-out effect on the foaming agent,^23,24^ and anionic surfactants precipitate in the presence of inorganic salt ions, preventing foam formation.^25,26^ Therefore, developing a new inorganic salt-enhanced inhibitory foam is of great significance in enhancing the foam stability and inhibitory effect.

On the other hand, single physical inhibitors possess oxygen barrier cooling effects but usually exhibit several shortcomings such as short duration of action and limited inhibitory effect. Single chemical inhibitors prevent the spontaneous combustion of coal by blocking the chain reaction of coal oxidation, which also has many disadvantages such as poor fire suppression and slow reaction times.^27−30^ A physicochemical composite inhibitor may alleviate the shortcomings of single physical and chemical inhibitors to achieve better inhibitory effects of coal spontaneous combustion. Melatonin (MLT), a generalized abiotic stress regulator in plants acting as a chemical inhibitor, can not only enhance the stability of the foam structure by providing electron-fixed free radicals but also reduce the number of active groups on the surface of the coal molecule.^31−33^ Such properties make MLT desirable for compounding with an inorganic salt inhibitor to form a physicochemical composite inhibitor, which is expected to further improve the inhibitory performance for coal spontaneous combustion.

In this study, four different inorganic salts, NaCl, CaCl_2_, Na_2_SO_4_, and NaHCO_3_, were selected as physical inhibitors. The effect of different inorganic salts on foam performance was studied to screen the optimal physical inhibitor. Furthermore, MLT in different ratios was introduced into the optimal inorganic salt solution to prepare a physicochemical composite inhibitor. The foam performance, wettability, and inhibitory performance of the composite inhibitory foam for coal spontaneous combustion were systematically investigated.

## 2 Materials and Methods

### 2.1 Materials

The sodium chloride (NaCl, AR), calcium chloride (CaCl_2_, AR), and melatonin (MLT, 98%) were purchased from Tianjin Hengxing Chemical Reagent Manufacturing Co., Ltd., Shanghai Puqian Optical Instrument Co., Ltd., and Aladdin (China), respectively. The sodium sulfate (Na_2_SO_4_, AR) and sodium bicarbonate (NaHCO_3_, AR) were purchased from Sinopharm Chemical Reagent Co., Ltd. Distilled water was used in the experiment. The coal samples used were medium-volatile bituminous coal, which were purchased from Ningxia Wangwa Coal Co., Ltd. This type of coal, characterized by high carbon content and high calorific value, is prone to spontaneous combustion and exhibits complex coal–oxygen reactions. It is used as a typical coal sample to validate the inhibitory effect of inhibitory foam in this work. Table 1 presents the detailed industrial analysis results of the coal sample.

**Table 1:** Detailed Industrial Analysis Results of the Coal Sample^a^

sample	*M*_ad_ (%)	*V*_ad_ (%)	*A*_ad_ (%)	FC_ad_ (%)
raw coal	13.00	32.79	4.95	49.26

a *M*_ad_—moisture content; *V*_ad_—volatile content; *A*_ad_—ash content; FC_ad_—fixed carbon content.

### 2.2 Preparation of Composite Inhibitory Foam and Coal Sample

#### 2.2.1 Preparation of the Composite Inhibitory Foam

First, the composite inhibitors were prepared by mixing selected CaCl_2_ inorganic salts and MLT in ratios of 6:1, 4:1, 2:1, and 1:1 by mass under continuous stirring for 15 min. Moreover, according to the foundation of previous experiments, the optimal composite foaming agent was prepared from lauryl acyl propyl betaine (LAB) and sulfonate (AOS) foaming agent in the ratio of 7:3, and the total concentration was 8 g/L. The composite inhibitory foam was prepared by stirring the composite inhibitors of different concentrations (5, 10, 15, 20 wt %) with the composite foaming agents.

#### 2.2.2 Preparation of the Coal Samples for Testing

The coal samples were prepared by mixing two coals with particle size ranges of 40–80 and 120–180 mesh in a 1:1 ratio by mass. The mass of each group of coal samples was set to 40 g, and the mass ratio of raw coal to composite inhibitory foam and water-based foam was 3:1. The raw coal for comparison and the inhibitory coal sample were subsequently dried at 30 °C for 24 h for later use.

### 2.3 Characterization

#### 2.3.1 Measurements of Foaming Ability, Foam Half-Life, and Surface Tension

A 50 mL foam solution was prepared in a 2000 mL plastic beaker and stirred at 2800 r/min for 5 min. The highest level of foam observed was recorded as the initial foam volume, characterizing the foaming capacity of the foam. After pouring the foam into a measuring beaker, the time taken for the foam volume to decrease by half was recorded as the volume half-life, serving as a measure of foam stability. The surface tension of the solution was determined by measuring the wetting force on a platinum sheet using a K100-type tensiometer (KRUSS company, Germany). The instrument could automatically perform five measurements, and the average value was taken. The experimental temperature was maintained at 25 ± 2 °C.

#### 2.3.2 Measurements of Droplet Contact Angle

Raw coal and KBR powder were mixed in a ratio of 1:200 to make thin slices for measurements. Droplet contact angle tests were carried out on coal samples using a DSA30 measuring instrument (KRUSS, Germany). Video recordings of droplet contact with the sample surface were made from 0 to 10 s, with instantaneous images captured at 2 s intervals.

#### 2.3.3 Measurements of Coal Spontaneous Combustion Characteristics

A Coal Spontaneous Combustion Characteristic Analyzer (GC-4175, East & West Analytical Instruments, Beijing) was used in conjunction with a gas chromatograph (GC4000A) to perform a programmed heating experiment. The experimental carrier gas was air with a flow rate of 100 mL/min, and the heating temperature ranged from 30 to 200 °C. Each time the coal samples were elevated by 10 °C, the generated gas was collected and then injected into the gas chromatograph to record the CO release.

#### 2.3.4 Measurements of Thermogravimetric Characteristics

The TG and DTG curves of coal during thermal decomposition were analyzed using a TGA3+ simultaneous thermal analyzer (Mettler Toledo, USA) to study the thermal characteristics of the raw coal and the inhibitory coal. The experimental carrier gas was air with a flow rate of 50 mL/min, and the heating temperature ranged from 30 to 800 °C with a heating rate of 10 °C/min.

#### 2.3.5 Microstructure of Composite Inhibitory Foam and Coal Sample

The microstructures of the raw coal and the inhibitory coal were measured by scanning electron microscope (FlexSEM1000, Hitachi Company, Japan). The acceleration voltage was set to 15 kV to examine the morphology of coal. Energy analysis observations were conducted using energy dispersive X-ray spectroscopy (EDS, U.K.).

## 3 Results and Discussion

### 3.1 Effect of Inorganic Salts on Foam Performance

**Figure 1:**
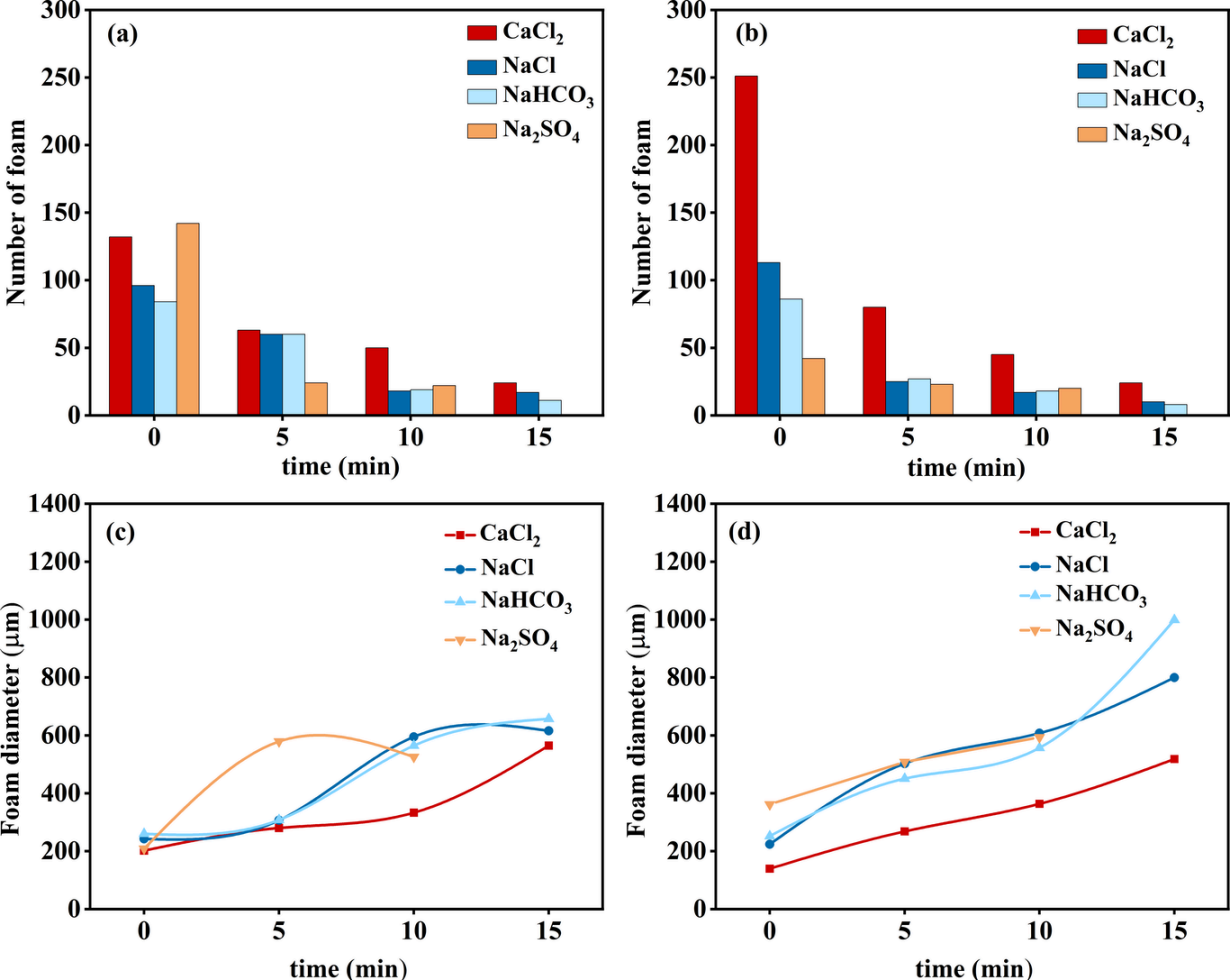
(a, b) Number of foams and (c, d) foam diameter containing (a, c) 2 g/L and (b, d) 5 g/L of inorganic salts as a function of time.

To investigate the effect of various inorganic salts on the foam performance, saline solutions with concentrations of 2 and 5 g/L containing NaCl, CaCl_2_, Na_2_SO_4_, and NaHCO_3_ were prepared, respectively. Figure 1a and b shows the number of foams containing 2 and 5 g/L of inorganic salts as a function of time. Clearly, after 15 min, the number of foams was 24 for both 2 and 5 g/L of CaCl_2_, while the foam with Na_2_SO_4_ completely collapsed. The numbers of the other two inorganic salt-based foams, NaCl and NaHCO_3_, were both less than 20. Figure 1c and d shows the variation of foam diameter with time for foams containing 2 and 5 g/L of inorganic salts. The initial foam diameters for CaCl_2_, NaCl, NaHCO_3_, and Na_2_SO_4_ at 2 g/L were 202.1, 242.5, 260.2, and 208.4 μm, respectively. Among the four types of inorganic salts added, CaCl_2_ resulted in the smallest foam diameter. Therefore, the addition of CaCl_2_ contributed to the formation of dense and uniform foams, slowing down gas diffusion and thinning of the liquid film by hindering excretion at the liquid film. This suppressive effect prevented bubble coalescence and enhanced the stability of the foam. When the concentration of CaCl_2_ was increased from 2 to 5 g/L, the initial bubble diameter decreased significantly, indicating the enhanced suppressive effect on bubble coalescence. Moreover, the growth rate of foam diameter with time slowed as the concentration of CaCl_2_ increased. Therefore, it can be inferred that CaCl_2_ has a more pronounced suppressive effect on foam polymerization.

**Figure 2:**
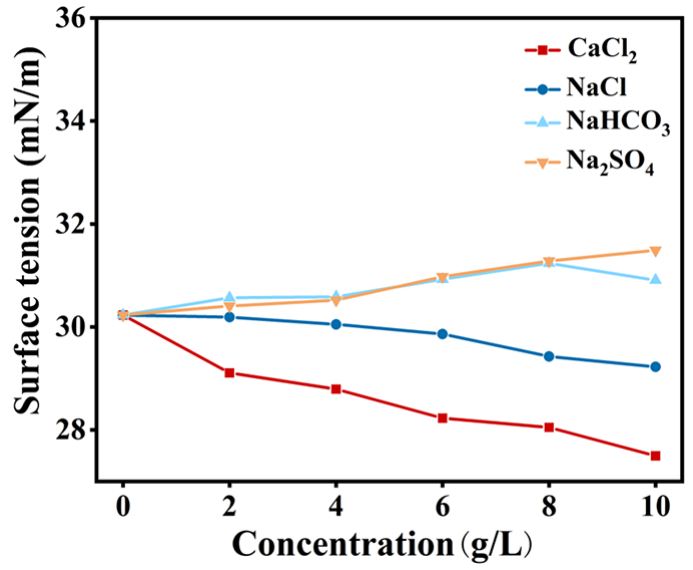
Variation of surface tension of the foaming agent solution with inorganic salt concentration.

The surface tension of the foaming agent solution was also measured at concentrations of 2, 4, 6, 8, and 10 g/L of the inorganic salts. Figure 2 shows the variation of surface tension of the foaming agent solution with inorganic salt concentration. It can be observed that the surface tension values of the solutions containing NaHCO_3_ or Na_2_SO_4_ gradually increased with the increasing concentration of inorganic salt. The NaHCO_3_- and Na_2_SO_4_-based foaming agent solutions reached the maximum surface tension values of 31.238 and 31.488 mN/m at and 10 g/L, which increased by 3.3% and 4.2%, respectively, compared to the water-based foams. On the contrary, the surface tension values of NaCl- and CaCl_2_-based foaming agent solutions decreased with increasing concentration, and the rate of decrease was more pronounced for CaCl_2_. As the concentration of CaCl_2_ increased from 2 to 10 g/L, the surface tension decreased from 29.108 to 27.496 mN/m. The variation of surface tension for CaCl_2_-based foaming agent solutions may be attributed to the counterions in CaCl_2_ neutralizing the charge of some hydrophilic groups in the blowing agent, thus reducing the interfacial potential and lowering the surface tension value.

From the foam diameter, number, and surface tension characteristics of different inorganic salt-based foaming agent solutions, it can be concluded that CaCl_2_ showed a pronounced suppressive effect on foam polymerization and excellent foam stability. Therefore, CaCl_2_ was chosen as a physical inhibitor for preparing the physicochemical composite inhibitor with MLT.

### 3.2 Determination of the Optimal Ratio and Concentration of CaCl_2_–MLT Composite Inhibitor

**Figure 3:**
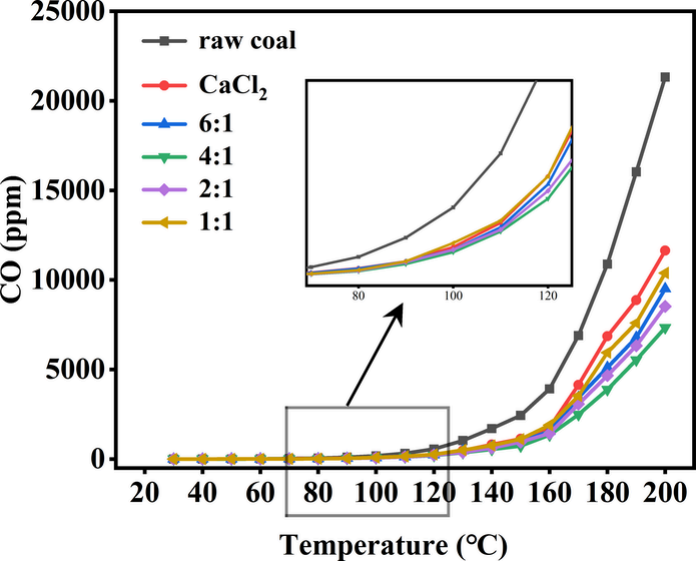
Temperature dependence of CO concentration released from coal samples containing different ratios of composite inhibitors and raw coal samples.

To investigate the optimal CaCl_2_–MLT composite inhibitor for inhibiting coal spontaneous combustion, we compared the release of CO in raw coal samples and treated coal samples by composite inhibitors of different ratios. Figure 3 shows the temperature dependence of CO concentration released from coal samples containing different ratios of composite inhibitors and raw coal samples. Obviously, from 30 to 80 °C, the CO release concentrations were all relatively low, with CO release concentrations below 100 ppm. The slow process of spontaneous combustion and oxidation of coal at this temperature stage resulted in minimal gas production. From 80 to 120 °C, the CO release concentration of the raw coal, CaCl_2_-treated coal sample, and 1:1 ratio of composite inhibitor with a treated coal sample started to increase. The increase in CO release concentration of the coal sample treated with the composite inhibitor was significantly lower than that of the raw coal. From 130 to 200 °C, the CO concentration released from all six coal samples increased markedly, and the increase of CO release in the raw coal was much higher than that in the composite inhibitor-treated coal samples. In this temperature range, the accelerated accumulation of heat in the raw coal led to intense coal–oxygen reactions and the generation of large amounts of gas. Especially, the concentration of CO in coal samples showed an exponential increase after reaching a temperature of 160 °C. However, the CO concentration produced by the inhibitory coal was significantly lower than that produced by the raw coal. This can be attributed to the presence of a large amount of water in the composite inhibitory foam. As the temperature rose, the composite inhibitory foam ruptured and released water into the coal, effectively reducing its temperature. Moreover, the composite inhibitory foam exhibited strong water absorption performance and formed a water film on the surface of the coal, preventing it from coming into contact with oxygen. During coal oxidation, Cl^–^ underwent substitution reactions with methyl and methylene groups, which increased the stability of ether and other surface structures.^34^ Ca^2+^ formed coordination compounds with active groups in the coal, preventing oxygen from complexing with −COO– in coal molecules. Additionally, MLT could increase the water retention of CaCl_2_, and the oxidation resistance of CaCl_2_ was enhanced. Compared to the coal sample treated with only CaCl_2_, the concentration of CO release from the coal samples treated with composite inhibitors decreased by 10.8% (1:1 ratio), 26.8% (2:1 ratio), 37.0% (4:1 ratio), and 18.4% (6:1 ratio) at 200 °C, respectively. When the mass ratio of CaCl_2_ to MLT was 4:1, the lowest CO release concentration of 7337.06 ppm was observed in the treated coal samples, indicating that the treatment of coal samples with the composite inhibitor of a 4:1 ratio could inhibit spontaneous combustion and oxidation of coal most efficiently.

**Figure 4:**
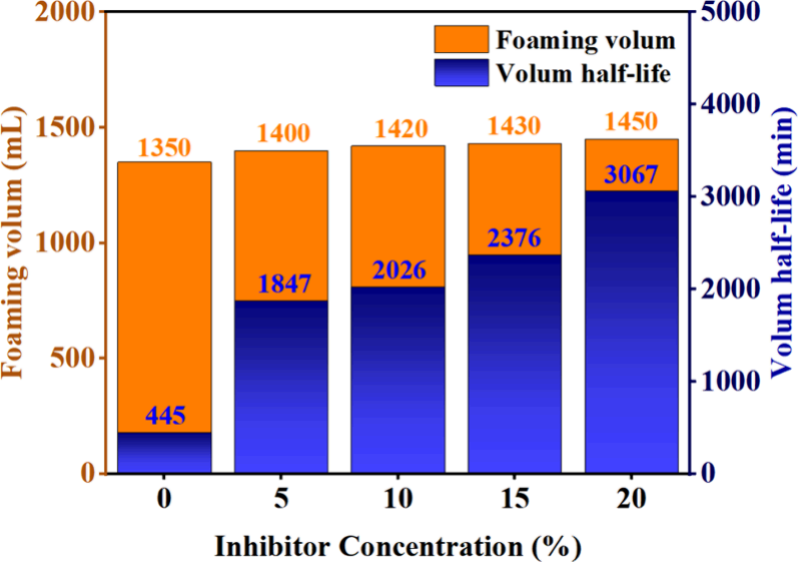
Foam performance at different concentrations of the composite inhibitor.

To determine the optimal concentration of the composite inhibitory so that the prepared composite inhibitory foam can better cover the coal seam, the effects of different concentrations of the composite inhibitor on the foaming ability and stability of the composite foaming agent were studied. Figure 4 shows the foam performance at different concentrations of the composite inhibitor. When the composite inhibitor was added at 0, 5, 10, 15, and 20 wt %, the foaming volumes of the inhibitory foam were 1350, 1400, 1420, 1430, and 1450 mL, respectively, and the corresponding volume half-lives were 445, 1874, 2026, 2376, and 3067 min, respectively. When the mass fraction of the composite inhibitor was too high, the interface molecules reached saturation, and the foaming ratio became stable. As a result, the half-life of the foam was greatly extended by the addition of the composite inhibitor. When 20 wt % of the composite inhibitor was added, the stability of the composite inhibitory foam increased from 445 min (water-based foam) to 3067 min.

### 3.3 Performance of the Composite Inhibitory Foam

**Figure 5:**
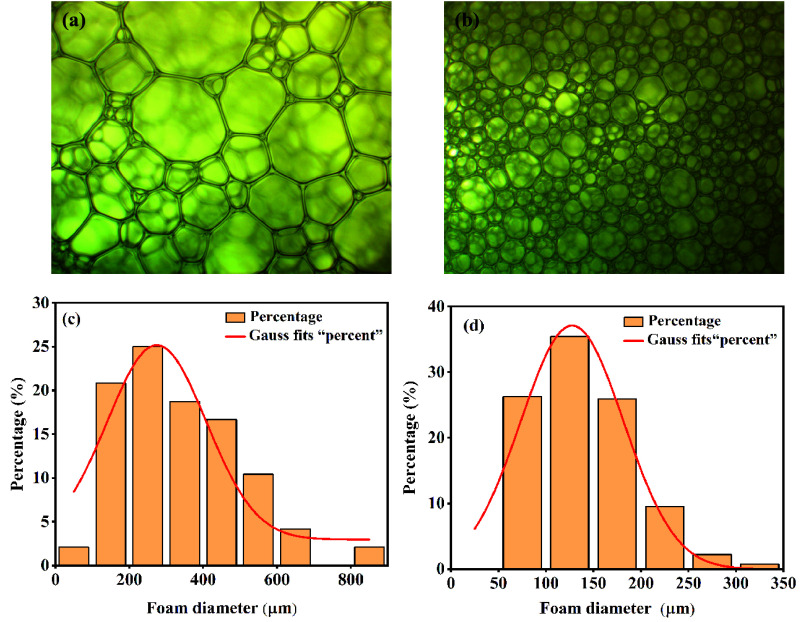
Microstructures and percentages of foam diameter distribution of the (a, c) water-based foam and (b, d) composite inhibitory foam.

The performance of the composite inhibitory foam based on 20 wt % of CaCl_2_–MLT composite inhibitor was further investigated systematically. Figure 5a and b shows the microstructures of the water-based foam and the composite inhibitory foam. In the water-based foam structure, the foam diameter distribution was not uniform, resulting in large differences in diameters. The maximum and minimum diameters were 819.9 and 83.8 μm, respectively, with a difference of 736.1 μm (Figure 5a). In contrast, the foam cells of the composite inhibitory foam were more numerous and uniform in size. The difference between the maximum (340.3 μm) and minimum (50.1 μm) diameters was 290.2 μm (Figure 5b). Figure 5c and d displays the foam diameter distributions of the water-based foam and the composite inhibitory foam. The average diameter of the composite inhibitory foam was 139.1 μm, with more than 60% of foam cells ranging from 50 to 200 μm in diameter. The addition of the composite inhibitor facilitated the inhibition of gas diffusion between foam cells, thereby enhancing the stability of the foam. This was attributed to the effect of Ca^2+^ on compacting the foaming agent molecules, which reduced the hydration of the head groups. Additionally, MLT can form complexes with Ca^2+^, effectively improving the foam stability.

**Figure 6:**
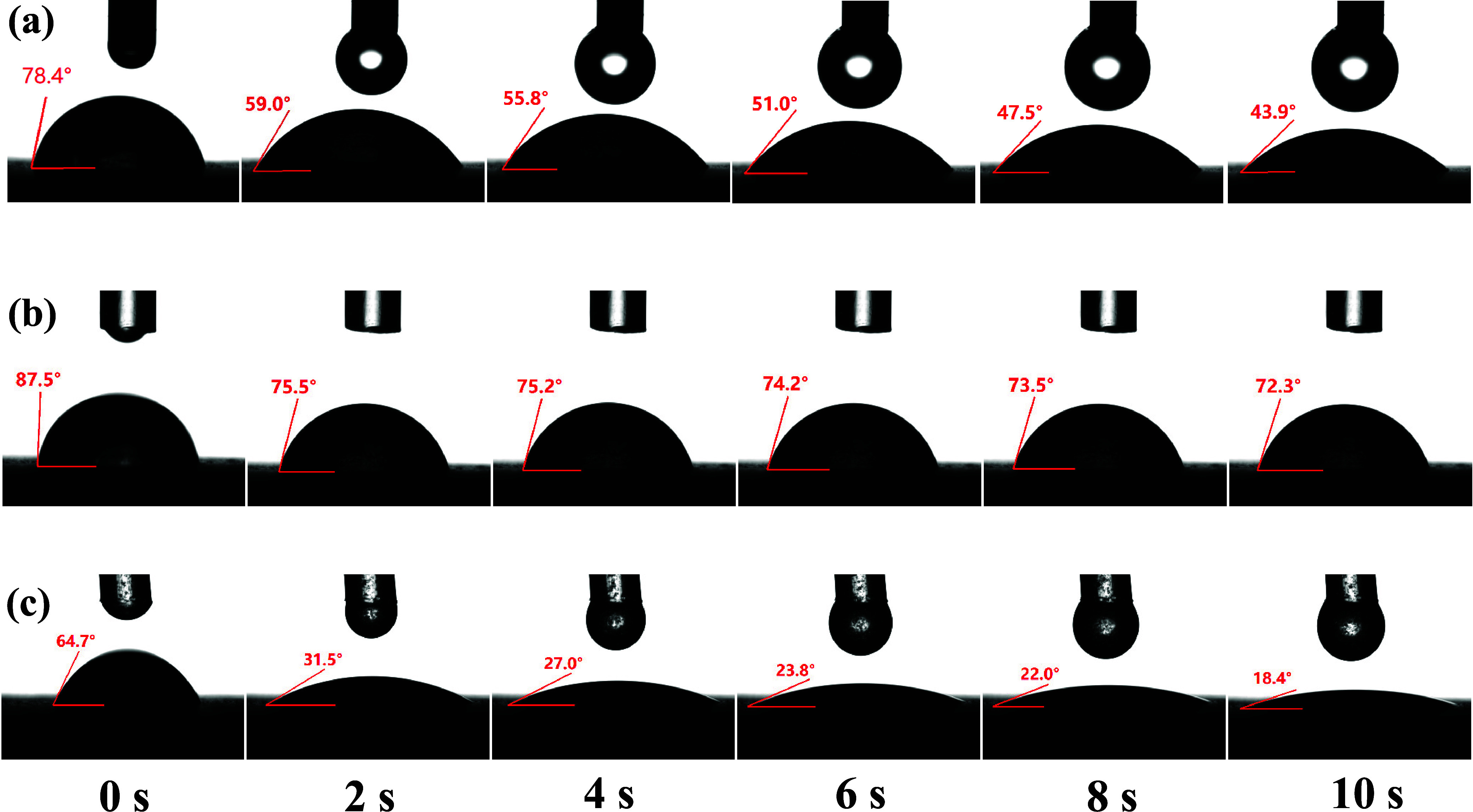
Contact angles formed by (a) pure water, (b) CaCl_2,_ and (c) the composite inhibitory foam on the coal surface.

Figure 6 shows the contact angles formed by pure water, CaCl_2_, and the composite inhibitory foam on the coal surface. Obviously, during the first 0–10 s, the contact angle decreased from 78.4° to 43.9° for water, from 87.5° to 72.3° for the CaCl_2_ solution, and from 64.7° to 18.4° for the CaCl_2_–MLT composite inhibitory foam solution. The contact angle of the three solutions decreased the most at 2 s. With the increase of time, the solution gradually spread on the coal samples, which increased the contact area between the solution and the coal samples and resulted in a slow decrease in the contact angle. Among the three coal samples, the CaCl_2_ solution had the largest contact angle, which was 72.3° at 10 s. This suggested that the CaCl_2_-based foam had relatively poor wetting ability and took a long time to penetrate the coal seam. Due to the effect of surface tension, the liquid always tends to contract into a spherical shape, reducing the contact area between the solution and the solid surface, which is not conducive to suppressing low-temperature coal oxidation. The composite inhibitory foam solution can effectively reduce the surface tension of the CaCl_2_ solution. Therefore, the contact area between the composite inhibitory foam solution and the coal sample increased, the contact angle decreased, and the wetting ability of the coal improved. The CaCl_2_–MLT composite inhibitory foam solution can penetrate into the coal body more effectively and suppress the low-temperature oxidation reaction of coal.

**Figure 7:**
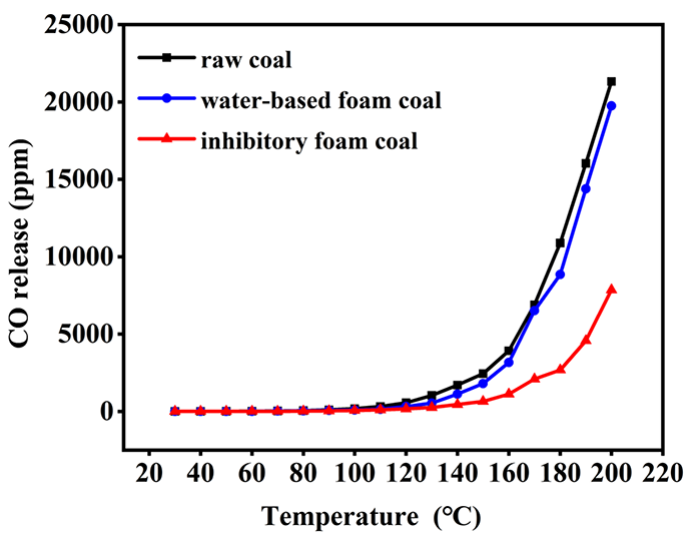
Variation of CO release concentration as a function of heating temperature in the raw coal, water-based foam coal, and inhibitory coal samples.

To investigate the inhibitory effect of the composite inhibitory foam, the volume fraction of CO released during the coal spontaneous combustion heating process was evaluated to analyze the inhibitory performance.^35,36^Figure 7 shows the variation of CO release concentration as a function of heating temperature in the raw coal, water-based foam coal and inhibitory coal samples. It can be observed that the concentration of CO release from the coal samples all first increased slowly and then rapidly. Raw coal started to produce CO at a temperature of 30 °C. After reaching 90 °C, the concentration of CO increased exponentially with increasing temperature. A large number of active functional groups in the coal were activated at 120 °C, leading to an accelerated chemical reaction rate and a rapid increase in CO release. Compared to the raw coal sample, both water-based foam coal and inhibitory coal sample exhibited lower CO concentrations before 120 °C. Above 120 °C, the concentration of CO release from all three coal samples increased exponentially, but this increase was significantly suppressed by the addition of CaCl_2_–MLT composite inhibitory foam. The CO release concentrations at 200 °C were 21326, 19745, and 7854.6 ppm for raw coal, water-based foam coal, and inhibitory coal, respectively, indicating that the composite inhibitory foam can inhibit the CO release more effectively than the water-based foam. These results were mainly attributed to the differences between the properties of water-based and composite inhibitory foams. A large amount of water contained in the foam would rupture and release the water into the coal, effectively lowering the temperature of the coal and retarding the oxidation reaction. However, water evaporated at high temperatures (>100 °C), causing the water-based foam to lose its inhibitory effect. In contrast, the CaCl_2_–MLT composite inhibitory foam was capable of covering the coal samples and converting into an inhibition solution to penetrate into the pores of the coal during the heating process, reducing the contact area between the coal and the air and thus slowing down the rate of oxidation of the coal and reducing the release of CO.

### 3.4 Thermogravimetric Characteristics of Coal Samples before and after Inhibition

**Figure 8:**
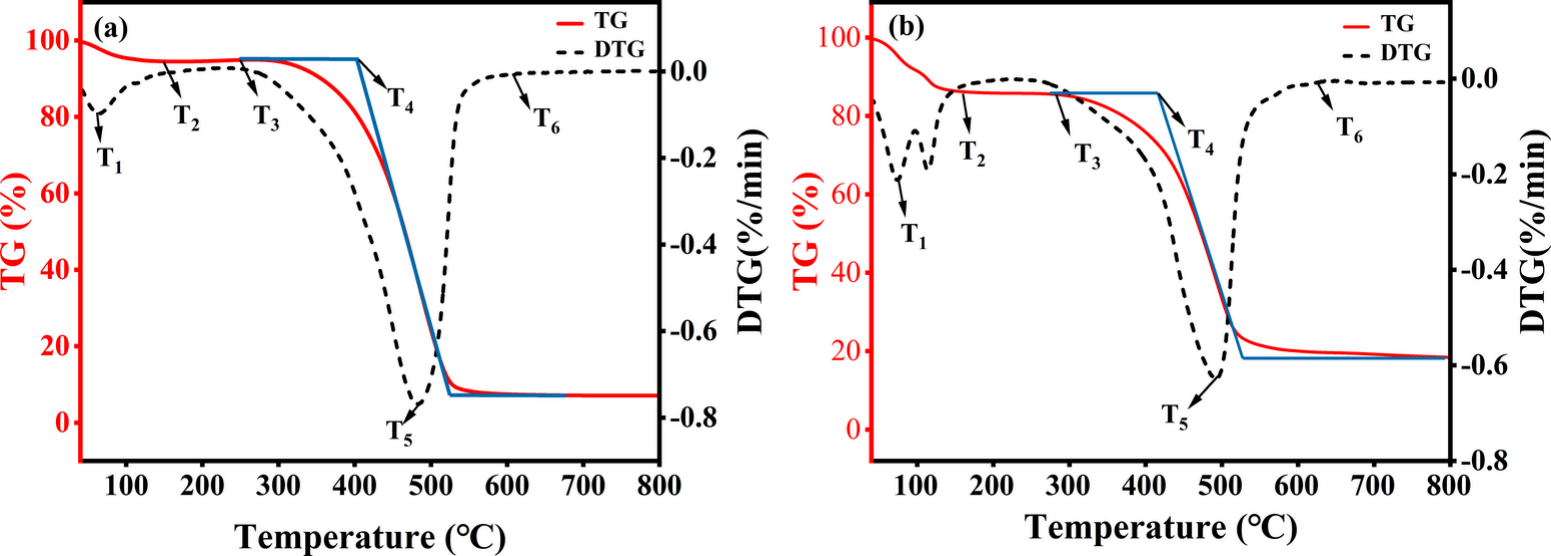
TG-DTG curves of (a) raw coal sample and (b) inhibitory coal sample.

To shed light the thermal stability and thermal decomposition characteristics of the raw coal and inhibitory coal, thermogravimetric (TG) analysis experiments were conducted. Figure 8 shows the TG-DTG (thermogravimetric-derivative thermogravimetric) curves of the raw coal and the inhibitory coal samples, where the DTG represents the derivative TG. The TG-DTG curves of coal contained six characteristic temperature points.^37^Table 2 lists the six characteristic temperature points (*T*_1_–*T*_6_) of the two coal samples, and Table 3 presents a division of the spontaneous combustion process of the two coal samples into temperature stages based on these characteristic temperature points. *T*_1_ represents the temperature at which the maximum water loss rate occurs, accompanied by significant water evaporation and gas adsorption–desorption, leading to an increase in mass loss. *T*_2_ represents the temperature at which the mass fraction of coal reaches a minimum after the desorption of water evaporation and adsorption gas in coal. This signifies the end of water evaporation and the beginning of oxygen absorption and weight gain. *T*_3_ represents the end of the oxygen absorption and weight gain phase of coal, initiating the pyrolytic oxidation of the aromatic structure in the coal. *T*_4_ is the temperature at which the mass reduction rate of the coal sample accelerates to the maximum weight loss rate. At this temperature, the aromatic structure of the coal is destroyed and participates in the oxidation reaction, intensifying coal combustion. *T*_5_ denotes the temperature point of the maximum thermal weight loss rate, indicating the peak combustion rate of coal samples. Finally, *T*_6_ signifies the temperature at which the mass of the coal sample stabilizes after combustion.

**Table 2:** Characteristic Temperature Points of Coal Samples

samples	*T*_1_ (°C)	*T*_2_ (°C)	*T*_3_ (°C)	*T*_4_ (°C)	*T*_5_ (°C)	*T*_6_ (°C)
raw coal	61.7	149.8	249.9	406.2	481.6	610.4
inhibitory coal	74.6	161.3	281.3	417.8	491.1	624.3

**Table 3:** Stage and Mass Loss of the Coal Spontaneous Combustion Process

	raw coal		inhibitory coal	
stages	temperature (°C)	TG changes (%)	temperature (°C)	TG changes (%)
water evaporation and adsorption	30.0–149.8	5.5	30.0–161.2	13.9
oxygen absorption and weight gain	149.8–249.9	–0.4	161.2–281.3	0.7
decomposition phase	249.9–406.2	15.4	281.3–417.8	13.1
combustion phase	406.2–610.4	72.1	417.8–624.3	52.7

Compared to the raw coal, the mass loss of T1 in inhibitory coal had more mass lost, and the *T*_1_ temperature of the inhibitory coal sample was delayed by 12.9 °C, indicating that the composite inhibitory foam increased the thermal decomposition temperature of small molecular compounds and active functional groups by facilitating moisture evaporation and heat absorption. Additionally, the composite inhibitory foam can inhibit the oxidation of molecular hydrogen bonding interactions and free hydroxyl, −CH_3_/-CH_2_–, and C–O/C–O–C functional groups.^38^ As shown in Table 2, the inhibitory coal sample underwent a significant thermal weight loss reaction, and the weight loss rate in the moisture evaporation and adsorption stage was 2.52 times larger than that of the raw coal. In addition, there was a noticeable difference in the oxygen absorption and weight gain stage (*T*_2_–*T*_3_) between the two coal samples. The temperature range differences were 100.1 °C for the raw coal and 120 °C for the inhibitory coal, respectively. With an increase in temperature, the highly activity functional groups in the raw coal made it highly susceptible to spontaneous combustion, resulting in a very short duration of the oxygen absorption and weight gain stage. In the inhibitory coal, the composite inhibitory foam not only enabled the coal samples to continuously decompose large molecular structures and form more high-activity functional groups during the oxidation and heating process but also adsorbed oxygen and released the accumulated heat, thus prolonging the duration of the process. As a result, the ignition temperature of the inhibitory coal sample was significantly higher than that of the raw coal. During the *T*_4_–*T*_6_ stage, the combustion and decomposition of aromatic ring structures led to a rapid decrease in the TG curves of the two samples above *T*_4_. It can be seen that the *T*_4_ and *T*_5_ temperatures of the inhibitory coal were higher than those of the raw coal (Table 1). The coal oxidation reaction ended at *T*_6_ temperature, beyond which the TG and DTG curves remained stable and unchanged. At this temperature point, the residual mass of the raw coal was 7.4 wt %, while that of the inhibitory coal was 19.7 wt %, which suggested that the inhibitory coal was more difficult to burn completely.

### 3.5 Microstructural Changes of Coal Samples before and after Inhibition

**Figure 9:**
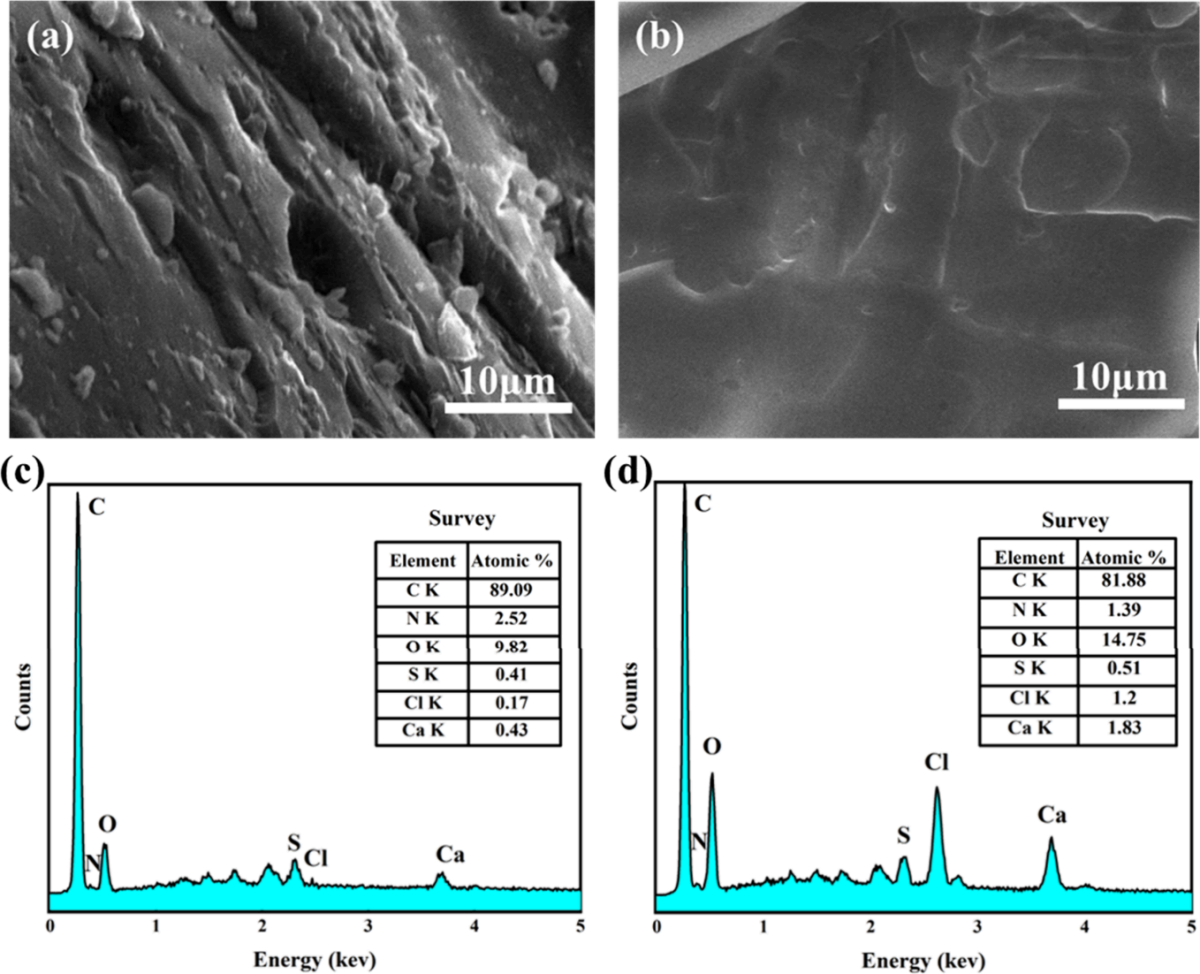
Surface (a, b) SEM images and (c, d) EDS patterns of (a, c) raw coal and (b, d) inhibitory coal.

The penetration effect of the composite inhibitory foam on coal can be evaluated by testing the microstructural changes of coal samples before and after the composite inhibitory foam treatment.^18,30^Figure 9 shows the SEM images of the raw coal and the inhibitory coal samples. As can be seen from Figure 9a, the surface of the raw coal was rough and contained numerous cracks and pores, which increased the contact area between coal and oxygen and thus accelerated the rate of coal oxidation. Moreover, the air trapped in the cracks and pits can store the heat generated during the coal oxidation, making the coal more prone to spontaneous combustion. By comparison, the surface of the inhibitory coal possessed a compact and smooth appearance, characterized by minimal coal debris or pores (Figure 9b). It can be inferred from the SEM images that the composite inhibitory foam has successfully penetrated into the coal samples through the coal pores and fracture structure, achieving coverage and encapsulation of the coal. This would prevent the oxygen from reaching the surface of the coal, reducing the adsorption of oxygen in the cracks of the coal and lowering the oxidation degree of the coal. The changes in the types and quantities of elements in the coal sample treated with the composite inhibitory foam can be observed through EDS, as depicted in Figure 9c and d. The raw coal surface primarily consisted of carbon and oxygen elements, with a small amount of calcium metal. However, after the composite inhibitory foam treatment, there was a significant increase in the detected content of oxygen, chlorine, sulfur, and calcium elements. This increase was attributed to the influence of AOS, LAB, and CaCl_2_ in the inhibitory foam, which was consistent with the SEM results.

## 4 Conclusion

To enhance the stability and inhibitory performance of the composite inhibitory foam for coal spontaneous combustion, a novel physicochemical composite inhibitor was developed in this work. To determine the best inorganic salt physical inhibitor, the effect of inorganic salts (NaCl, Na_2_SO_4_, NaHCO_3_, and CaCl_2_) on foam performance was studied. The smallest average diameter, slowest growth rate, and lowest the surface tension values were obtained by the CaCl_2_-based foaming agent solutions, indicating good foam stability. A physicochemical composite inhibitor based on CaCl_2_–MLT was prepared. When the mass ratio of CaCl_2_ to MLT was 4:1, the lowest CO release concentration of 7337.06 ppm at 200 °C was observed in the composite inhibitor-treated coal. Furthermore, the addition of 20 wt % of the composite inhibitor resulted in a foam half-life of 3067 min, which was 5.89 times longer than that of the water-based foam. In comparison with the water-based foam, the composite inhibitory foam based on 20 wt % CaCl_2_–MLT composite inhibitor exhibited more excellent foam stability, wetting ability, and inhibition performance. The release of CO at 200 °C was 7854.6 ppm, showing a reduction of 63.2% compared to the raw coal. Moreover, the composite inhibitory foam could significantly delay the onset of the characteristic temperature and reduce the weight change during the decomposition stage by 12.8%. SEM analysis of coals before and after inhibition suggested that the composite inhibitory foam has successfully penetrated into the coal samples, achieving coverage and encapsulation of the coal and lowering the oxidation degree of the coal. This work provides a new way to prevent spontaneous combustion and brings potential economic and safety benefits to the industry. In our future work, the inhibitory effect of the foam system on different coal types and the applicability of inhibitory foam system in real coal mining environments need to be further studied.
